# Community and health systems barriers and enablers to family planning and contraceptive services provision and use in Kabwe District, Zambia

**DOI:** 10.1186/s12913-018-3136-4

**Published:** 2018-05-31

**Authors:** Adam Silumbwe, Theresa Nkole, Margarate Nzala Munakampe, Cecilia Milford, Joanna Paula Cordero, Yolandie Kriel, Joseph Mumba Zulu, Petrus S. Steyn

**Affiliations:** 1 Department of Health Policy and Management, School of Public Health, University of Zambia, P.O Box 50110, Lusaka, Zambia; 2 Department of Obstetrics and Gynaecology, University Teaching Hospital, Lusaka, Zambia; 3 Department of Obstetrics and Gynaecology, MatCH Research Unit (MRU), Faculty of Health Sciences, University of the Witwatersrand, Durban, South Africa; 4 Department of Reproductive Health and Research, World Health Organisation, Geneva, Switzerland; 5 Department of Health Promotion and Education, School of Public Health, University of Zambia, P.O Box 50110, Lusaka, Zambia

**Keywords:** Barriers, Community, Contraception, Enablers, Family planning, Health system

## Abstract

**Background:**

Unmet need for contraception results in several health challenges such as unintended pregnancies, unwanted births and unsafe abortions. Most interventions have been unable to successfully address this unmet need due to various community and health system level factors. Identifying these inhibiting and enabling factors prior to implementation of interventions forms the basis for planning efforts to increase met needs. This qualitative study was part of the formative phase of a larger research project that aimed to develop an intervention to increase met needs for contraception through community and health system participation. The specific study component reported here explores barriers and enablers to family planning and contraceptive services provision and utilisation at community and health systems levels.

**Methods:**

Twelve focus group discussions were conducted with community members (*n* = 114) and two with healthcare providers (*n* = 19). Ten in-depth interviews were held with key stakeholders. The study was conducted in Kabwe district, Zambia. Interviews/discussions were translated and transcribed verbatim. Data were coded and organised using NVivo 10 (QSR international), and were analysed using thematic analysis.

**Results:**

Health systems barriers include long distances to healthcare facilities, stock-outs of preferred methods, lack of policies facilitating contraceptive provision in schools, and undesirable provider attitudes. Community level barriers comprise women’s experience with contraceptive side effects, myths, rumours and misconceptions, societal stigma, and negative traditional and religious beliefs. On the other hand, health systems enablers consist of political will from government to expand contraceptive services access, integration of contraceptive services, provision of couples counselling, and availability of personnel to offer basic methods mix. Functional community health system structures, community desire to delay pregnancy, and knowledge of contraceptive services are enablers at a community level.

**Conclusions:**

These study findings highlight key community and health systems factors that should be considered by policy, program planners and implementers in the design and implementation of family planning and contraceptive services programmes, to ensure sustained uptake and increased met needs for contraceptive methods and services.

**Electronic supplementary material:**

The online version of this article (10.1186/s12913-018-3136-4) contains supplementary material, which is available to authorized users.

## Background

Family planning and contraceptive programmes (FP/C) play a critical role in national and human development. They facilitate regulated population growth that results in social-economic benefits such as decreased poverty levels, enhanced education opportunities and reduced gender inequality [1]. Furthermore, they provide an opportunity for improved maternal and child health, through prevention of sexually transmitted diseases (STIs), unwanted and early pregnancies, and unsafe abortions [2]. Cates et al., state that FP/C programmes are among the most cost-effective development investments because of their direct influence on improving lives through national security and enhanced financial resources for communities [3].

The United Nations has prioritised increasing and sustaining utilisation of FP/C services as one of its eminent strategic investment focus areas in attaining sustainable development goals (SDGs) [4]. The target is to achieve universal access to sexual and reproductive health (SRH) services, including family planning, information and education by 2030 through investment in various FP/C services programmes at national and community levels [5]. FP/C services programmes have and continue to play a major role in raising the prevalence of contraceptive use globally. Cleland et al., [6] state that these programmes are responsible for raising contraceptive prevalence from less than 10% to 60%, and reducing fertility in developing countries from six to about three births per woman on average over the past six decades.

Despite these recorded improvements, FP/C services/method uptake still remains low in various parts of the world including Asia and Latin America but continues to be lowest in sub-Saharan Africa [7]. Approximately, 14 million unintended pregnancies occur in sub-Saharan Africa annually, and a large proportion are due to lack of access to FP/C services/methods [2]. As of 2015, an estimated 225 million women in developing countries would have preferred to delay or stop child bearing, but were not using any method of contraception [8]. Various reasons have been advanced for low uptake of these services such as limited method choices, limited access to contraception, poor quality services, users and provider bias and also gender-based barriers, particularly for marginalised groups like adolescents and the poorer sections of society [9].

In Zambia, the overall unmet need for contraceptives among married women stands at 21%, of which 14% are spacers and 7% are limiters [10]. The contraceptive prevalence rate (CPR) is at 47%, with a noticeable difference between rural and urban communities [10]. Many studies have focused on identifying and highlighting the complex social and economic factors at play during the supply of FP/C services [11–13]. However, less has been documented about community and healthcare providers’ perspectives regarding the underlying community and health system factors that shape provision and utilisation of FP/C services/methods.

The health system consists of various elements involved in healthcare delivery, including human resources for health, service delivery, and supply chain and governance systems [14]. At community level, the health system can be said to comprise “a set of local actors, relationships, and processes engaged in producing, advocating for, and supporting health in communities, but existing in relationship to established health structures” [15]. The interaction of community and health systems factors has a critical role to play in as far as the success or failure of a given health programme. This paper, therefore, seeks to document community and health systems barriers and enablers to provision and utilisation of FP/C services/methods.

## Methods

### Study design

This study was part of the formative phase of a multi-country (Kenya, South Africa and Zambia), complex designed intervention, to increase contraceptive met needs, through community and healthcare provider (HCP) participation in the provision and use of FP/Cs, within a human rights framework (the UPTAKE Project). The proposed intervention consisted of a facilitated community and HCP dialogue using a theory of change framework to identify, implement and evaluate activities (within the human rights domains), to increase contraceptive met needs. The project was commissioned by the World Health Organisation (WHO), and formative work was conducted between 2015 and 2016. It was designed to be executed in two phases, formative (intervention development) and intervention (implementation), respectively.

During the formative phase, we conducted a mapping of national family planning legislation and policies, facilities and services and exploratory qualitative research, which contributed to identifying key human rights domains, within which to contextualise the intervention focus areas and activities to increase met needs in each country. This study focuses on the qualitative research activities conducted in Zambia, which explored; (i) Knowledge, attitudes, and practices in FP/C services and utilisation; (ii) barriers and enablers to FP/C services; (iii) understanding of quality care and; (iv) community participation practices and activities (please see Additional files 1, 2, 3 and 4). For this manuscript, we report on the second objective (barriers and enablers to FP/C services).

### Data collection

A total of 14 focus group discussions (FGDs) were undertaken, each lasting between 60 to 90 min. Of these, 12 consisted of community members (users and non-users) and two were conducted with HCPs (stratified as managerial and frontline providers) (Table 1). Ten in-depth interviews (IDIs) lasting between 30 and 60 min were conducted with key stakeholders (Table 2). Both IDIs and FGDs were conducted by experienced facilitators who were supervised by the research team on best practices in qualitative research. The facilitators were selected from various backgrounds with relevant experience, including FP/C services providers, an expert on community participation approaches and an adolescent experienced in youth mobilisation. All had the essential understanding of local context and language.

**Table 1:** FGD participants

Focus group discussions (categories)	Age in years	Number of participants
Community members		
Females, urban, adolescents	15–19	10
Females, rural, adolescents	15–19	09
Females, urban young adults	20–34	08
Females, rural young adults	20–34	10
Females, urban adults	35–49	08
Females, rural adults	35–49	09
Females, unmarried young adults	20–34	10
Females, married young adults	20–34	10
Females, no-children	18–49	10
Males, adolescents	15–19	10
Males, young adults	20–34	10
Males, adults	35–49	10
Total		114
Healthcare providers		
Healthcare providers-managerial	–	10
Healthcare providers-frontline	–	09
Total		19
Total FGD participants		133

**Table 2:** IDI participants

Participants (categories)	Number of interviews
Political leadership	1
Neighbourhood health committee	1
Sexual and reproductive health non-governmental organisation	1
Traditional leadership	1
District health office	1
Provincial medical office	2
Teacher	1
Religious leadership	2
Total IDI participants	10

### Study setting

The study was conducted in Kabwe district, the provincial capital of the Central Province of Zambia, which has a population of 217,843 people, of which 58,381 (26.8%) are women in the reproductive age group of 15–49 years [10]. The district was chosen by both WHO and Zambian team of investigators due to its high unmet need for contraception [10]. After mapping of all health facilities in the district, a single health facility with a large catchment area catering for both rural and urban communities (peri-urban) was chosen as the study site for the qualitative research.

### Study participants and recruitment

The community members recruited for the FGDs were those within the reproductive age range (15–49 years). These were stratified into three male and nine female groups. All the FGD participants were selected and categorised into groups according to age - adolescents (15–19 years), young adults (20–34 years) and adults (35–49 years). Female groups were further categorised according to location (*n* = 6) -either rural or urban, marital status (*n* = 2) - either married or unmarried, and according to parity (*n* = 1) - those without children (Table 1).

Though purposive, the recruitment process of community members ensured a participatory approach by engaging a local district coordinator who was recommended by the District Health Office (DHO). The coordinator worked with the nursing sister-in-charge at the study site (selected health facility) together with some community health representative groupings, in recruiting participants for FGDs.

Recruitment of key informants for IDIs was also done through purposive sampling. This sampling technique ensured representation of key groups, and was based on knowledge of FP/C services experts, as well as key community persons who were influential in the health sector. Some of the key informant participant categories included: political, traditional and religious leadership, sexual and reproductive health non-governmental organisation representative, district and provincial medical officers, and a teacher from the education sector (Table 2).

### Data analysis

Data were analysed using thematic analysis, which is a method for identifying, analysing and reporting patterns (themes) within data [16]. Thematic analysis organises and describes the data set in (rich) detail and goes further to interpret various aspects of the research topic. The data analysis process started with the collection of information gathered through field notes. Interviews and FGDs were audio recorded in local language-Bemba, with participant permission, and transcribed and translated verbatim into English.

A qualitative data analysis software, NVivo (version 10, QSR International) was used to organise and manage the data. A single master code-list with thematic definitions was iteratively developed by researchers from the three countries. The researchers then double coded the transcripts to determine the validity of the cross-country coding, and to guide discussions around emergent themes, which arose from the study data. Any differences in coding were discussed within the qualitative research team. The master code-list was updated based on these discussions, and the codes were grouped into major and emergent themes. A consolidated NVivo database containing transcripts from the three countries was created. After every coding activity was completed, country NVivo databases were shared and merged to create a single database with comparable country data [17], which enabled country-specific data analysis.

### Ethical considerations

This study received WHO Ethics Review Committee (ERC) and Research Project Review Panel (RP2) approval. In addition, for the Zambian component of the study, ethical approval was obtained from the University of Zambia Biomedical Research Ethics Committee (UNZABREC) to conduct the research, and all prerequisite authorisations were obtained from the Ministry of Health. All participants (> 18 years) provided written, informed consent to participate in the study. Participants who were under the age of 18 years provided written assent, and their parents/guardians provided written consent for their participation. In the event that participants were not literate, a witness was required to be present during the consenting process and sign consent on their behalf. The participants gave separate consent to being audio recorded.

## Results

Every FGD was distinctively composed to allow for attribution of themes to specific groups as well as maximise participation within the discussions. Similarly, the IDIs included a variety of categories representing both community and health sector key stakeholders. No major differences were noted in the discussions by the different participant groups. However, where the views were specific to certain groups, these are noted in the manuscript. Below we present community members, HCPs and key stakeholders' perspectives regarding the factors hindering and enabling family planning and contraceptive services provision and utilisation, at health systems and community levels. Relevant quotes are provided to support each of the identified themes.

## Barriers to family planning and contraceptive services provision and utilisation

### Health systems barriers

#### Long distances to health facilities

Community participants from rural areas recounted that walking long distances to healthcare facilities in order to access FP/C services hindered utilisation. They narrated that long distances were demotivating to women who wanted to consistently use FP/C services/methods, and were a major contributor to discontinuation and intermittent use. The long distances also put clients at risk of being denied access to FP/C services if they got to the health facility outside the established schedule of service provision.

*“The problem I have seen is that some women live very far away and the time is fixed for provision of family planning and contraceptive methods such that when they want to use, they cannot access the services. They are told they are late and they should come next time. So this is another problem.” [Female FGD, Rural Young Adult, UZFG_RY003]*

#### Undesirable healthcare provider attitudes

Undesirable HCP attitudes were stated as a barrier to FP/C services utilisation, especially for marginalised user groups, like the unmarried and adolescent users. The healthcare providers and key community stakeholders reported that negative attitudes such as shouting, scolding, not allowing clients to explain their side effect experiences, and giving preference to socially accepted FP/C services user groups like the married women, existed in some of the health facilities.

*“To be frank some healthcare providers are rude, very rude. That is the major complaint. That is why even most women shun away, they prefer buying from drug stores in town instead of going to clinics.” [Laughs] [Key stakeholder IDI, traditional leader, UZI008]*

*“Youths in most cases when they go to the health facility to access family planning, the attitudes of caregivers send them away, especially when they are scolded to say, 'you’re still in school, you’re supposed to concentrate on books and not coming for family planning.'” [Healthcare provider FGD, Managerial, UZHG_L004]*

#### Stock-outs and lack of long acting reversible contraceptives

Stock-outs of preferred contraceptive methods and unavailability of long acting reversible contraceptives (LARCs) in some facilities, negatively affected contraceptive utilisation, as it meant that communities could not use nor access such services when they wanted to. Furthermore, it was reported that some healthcare facilities where unable to offer LARCs because some of the health personnel had not undergone training to provide these methods.

*“* *The pills should always be available at the clinic, not whereby whenever you go there, they tell you that they are not available every now and then. In the end, they even become rude to you and you also lose interest in going there” [Female FGD, Rural Young Adult, UZFG_RY002]*

*“I think the challenge like for the Intra-Uterine Device is that some facilities are unable to offer because some members of staff are not trained to offer that service.” [Key stakeholder IDI, Health Sector, UZI006]*

#### Lack of policies facilitating contraceptive provision in schools

The lack of policies aimed at facilitating contraceptive methods provision in schools was cited by some key stakeholders as a barrier to adolescent access to these services. The key stakeholders reported that adolescents were being denied an opportunity to receive the much-needed contraceptive services, despite the reported high levels of early marriages and teenage pregnancies in the district.

*“There should be a policy by the Ministry of Education that allows sexually active girls to access contraceptives in schools. We cannot stop these girls from using contraceptives because they are in school. If we do that, we will end up losing our teenage girls through unwanted pregnancies and criminal abortions.” [Key stakeholder IDI, Health Sector, UZI006]*

### Community level barriers

#### Women’s experiences with contraceptive side effects

All community members reported that the side effects of hormonal methods were a major barrier to using contraceptives. They cited prolonged and irregular menses, dizziness, headaches, stomach-aches, weight gain and weight loss as some of the most common side effects of contraceptive methods. These side effects were said to be the main reason as to why people discontinued, changed or stopped using particular contraceptive methods.

*“Like for me I used to take the pill safe plan* [name of contraceptive pill]*, it used to give me prolonged periods then I tried the injection for three months, I never used to see my periods, so I stopped. I prefer condoms.” [Female FGD, Married Young Adult, UZFG_MY001]*

#### Myths, rumours and misconceptions about contraceptive methods

Both community members and HCPs reported that a number of myths associated with various FP/C methods negatively affected community use of methods/services. The myths included concerns that FP/C methods could cause general body harm, impact on future births, cause infertility, and result in reduced sexual pleasure. Some respondents reported that taking the contraceptive oral pill (CoC) could result in giving birth to a lame child, for example, a baby with an abnormal head.

*“They say if you are taking the pill, your children will be lame, your children will grow up with big heads, and so on and whatever, trying to discourage people from taking the pills.” [Female FGD, Young Adult, UZFG_Y006]*

Others narrated that FP/C methods, such as CoCs like Mycrogynon 30 and Safe plan, could accumulate in the stomach, resulting in the development of fibroids and cancer of the stomach.

*“When you are taking pills like* Mycrogynon *and Safe plan, you are likely to develop cancer in the near future. So, you have to be going for cancer check-ups every now and then.” [Female FGD, Married Young Adult, UZFG_MY001].*

Injectable contraceptives like Depo-Provera were thought to result in delayed or difficulty in having future pregnancies. Furthermore, adolescents stated that myths were widely used by the elderly to discourage FP/C service use, for example, they were told that using FP/C methods at a young age could result in failure to conceive in future.

*“Some say if you are taking family planning you will be barren. You will not have a child in your life.” [Female FGD, Urban Adolescent, UZFG_UT007]*

#### Stigma towards certain user categories (adolescents and unmarried)

Societal stigma was cited by both community members and HCPs as one of the major hindrances to adolescent access and utilisation of FP/C services. Adolescents reportedly would rather not utilise FP/C methods/services, due to negative HCP attitudes and the risk of community members knowing that they were sexually active.

*“In this community it’s actually known that family planning services can only be accessed by married couples. The community does not expect a young person to access family planning. So the greatest barrier has been stigma and discrimination.” [Key stakeholder IDI, Health Sector, UZI004]*

Both adolescents and unmarried users reported being stigmatised when they accessed FP/C services, as FP was generally thought to be only for adults and married people.

*“The other experience I have gone through is that us who [are] unmarried, we are not usually free to be helped because at times they [healthcare providers] look down upon us when we come to collect the methods. They perceive us to be prostitutes or very promiscuous individuals.” [Female FGD, Unmarried young adult, UZFG_SY007]*

*“When you go to the clinic, the first question the nurse or doctor will ask is “are you married?” Then you if you say no, the next question will be, “but why do you want to use FP?” Yet you yourself know that you are sexually active.” [Female FGD, No children, UZFG_C008]*

#### Religious beliefs

Community members and HCPs narrated that certain religious beliefs were barriers to provision and use of FP/C services, because they discouraged people from using any method. Some religions believed that the use of contraception was synonymous to committing abortion, which is considered sinful. Additionally, provision of FP/C services to unmarried users was generally considered to be inappropriate as it was thought to be promoting promiscuity and sex before marriage in society.

*“We were discussing about family planning at our ministry and the issue that came out strong was that, when the egg is released and you use family planning medicine, it will make you abort. They said that the function of a pill is to abort the pregnancy each time you get pregnant. So, it is not right for us to abort because abortion is murder.” [Female FGD, Urban adult, UZFG_UA008]*

## Enablers to contraceptive services provision and utilisation

### Health systems enablers

#### Couples counselling services

Provision of counselling services before and after administering of FP/C methods in most heath facilities was reported by HCPs as an enabler. Couples counselling services in some facilities were also cited by HCPs as being particularly helpful in facilitating male involvement in FP/C services uptake and support.

*“We are promoting couples counselling in most of the health centres in Kabwe district. Not only are we targeting women alone, but also men as well. We encourage women to come together as couples with the men, so that when they want to access family planning, they understand what type of services we offer at facility level.” [Healthcare provider FGD, Frontline, UZHG_L007]*

#### Availability of personnel trained to offer the basic contraceptive method mix (barriers, short and medium acting)

Healthcare providers reported that personnel, in both urban and rural facilities, were trained to provide most of the basic contraceptive methods and services, like condoms, CoC pills (Safe plan and Mycrogynon 30) and injectables (Depo-Provera and Nuristerat). This was perceived to be an enabler, as it provided a basis for provision of the minimum method mix to the community members.

*“We are privileged as Kabwe, they trained most healthcare providers to provide at least most of the common and basic contraceptive methods. Three quarters of the health workers have been trained, though some do not practice. We also have mentors at the district level who mentor the providers at the facilities.” [Healthcare provider FGD, Frontline, UZHG_L002]*

#### Integration of FP/C services with other healthcare services

Healthcare providers reported that integration of services in the health facilities provided an enabling environment to reach as many clients as possible and provide them with FP/C services. The integrated model of service provision mentioned by most HCPs facilitated service provision, as it enabled clients who may have come for other healthcare services to be provided with FP/C services/methods.

*“In every clinic there is this integration of services. For example, if the client comes for antenatal we include services like family planning, if she needs to go for ART, the same day she will even go for ART to collect her ARVs. We don’t have to let the clients make trips to our clinics. When they come, it’s like a supermarket. They move from point B to point C, from point C to D, just like that.” [Healthcare provider FGD, Managerial, UZHG_H005]*

#### Government commitment to extending FP/C services

Key stakeholders from the health sector explained that there was renewed government commitment towards FP/C services provision in Kabwe district. They reported that the increased number of new health facilities and healthcare personnel being deployed to rural areas would improve access to contraceptive services. Additionally, it was stated that the provincial medical office had deliberately taken it upon themselves to strengthen FP/C services provision and use within the district, due to poor indicators, such as the low contraceptive prevalence rate (CPR) and high unmet need for contraception.

*“One of them is what the government is doing in opening up more health facilities, especially the health posts. This is contributing to reducing the physical distance that most women have to travel to access FP/Cs. Though there is still much more to be done, such efforts are contributing FP/C services being taken close to the people.” [Key stakeholder IDI, Health Sector, UZI007]*

### Community level enablers

#### Community-based health structures

Community participants from rural areas reported that the existing network of community-based health workers, like community based distributors (CBDs), traditional birth attendants (TBAs), neighbourhood health committees (NHCs) and safe motherhood action groups (SMAGs), facilitates FP/C services/method provision and utilisation. This network reportedly acts as a link between the community and the healthcare systems, providing a variety of healthcare services including FP/C methods/services to people, who otherwise may not be reached by the healthcare system.

*“We have what are called community-based distributors. These are based in the community and they are able to explain and also provide, especially the oral contraceptives.” [Female FGD, Rural Young Adult, UZFG_RY006]*

Other community structures included cooperatives, churches, community drama groups, football clubs, youth and women’s groups, and the savings groups which could also be explored to empower community members with FP/C services/methods information.

*“There are gatherings where community members meet. For example, saving groups, interdenominational prayers, and sometimes we have women meetings to share information, like for family planning. There are also some cooperatives where we share things for farming. So, there are a lot of situations which require us to meet as a community.” [Key stakeholder IDI, Community, Religious Leader, UZI009]*

#### Community desire to delay pregnancy

Community members from both urban and rural areas reported that they desired to delay pregnancy until they were financially and psychologically ready to support their babies. To this effect, they indicated that using FP/C methods and services allowed them to concentrate on work, complete their schooling, avoid unwanted pregnancies, and raise healthy and planned families.

*“We use family planning because most of us* [girls]*, we don’t want to get pregnant at an early age, so we propose to our partner to use a condom when we are having sex.” [Female FGD, Adolescent, UZMG_T005]*

*“It gives* [us a] *chance to work. Usually you stop work temporarily if you have a child. family planning allows you to plan so that you are not disturbed by work stoppages due frequent child bearing. You can plan that at least three years passes before having another child.” [Female FGD, Married Young Adult, UZFG_MY006]*

#### Community knowledge of FP/C services

Community members had knowledge of most of the common and basic FP/C methods, but had less knowledge of emergency contraceptives and other more advanced methods like LARCs. Some participants were able to define FP, others could explain how particular contraceptive methods worked, as well as their advantages and disadvantages. Community members also had knowledge of natural FP methods, which they used, including withdrawal, thigh sex and breastfeeding methods.

*“FP is a way a way of taking care of oneself where child bearing is concerned. A woman's body needs time to rest after delivery, say 2 to 3 years after delivery, after which one can be able to get pregnant again. The other thing I know is that it helps us to bring up our children well in terms of feeding, clothing and taking them to school.” [Female FGD, Urban Adult, UZFG_UA002]*

*“The methods of family planning we use in our rural areas, if one fails to go to the clinic and your husband comes, you let him ejaculate between your thighs or he withdraws and ejaculates from outside. It helps us until the day we go to get an injection for family planning.” [Female FGD, Rural Adult, UZFG_RA005].*

## Discussion

Since the UPTAKE Project sought to bring together community and health systems efforts in increasing met needs for contraception, these findings were of major importance in situating and understanding the context in which the proposed intervention would be implemented. Furthermore, these findings facilitated identification of bottlenecks and facilitators to contraceptive provision and use at both community and health systems levels to inform intervention activities to increase met needs. Below, we discuss the findings around the two core thematic areas of community and health systems barriers and enablers to family planning and contraceptive services provision and use.

### Barriers to family planning and contraceptive services provision and utilisation

While these findings underline government commitment to extend health services to remote areas, long distances to some health facilities still remain a challenge to access and use of contraceptive services in many rural areas. Community efforts to leverage this includes using local structures to distribute and generate demand for contraceptives. However, many of these local structures still face numerous challenges, such as lack of incentives (for example bicycles) to function optimally [18]. Appropriate incentives, as well as effective management strategies that enhance local structures’ capacity to deliver FP/C services can help increase met needs for contraception [19].

Though most Zambian policies on SRH do recommend provision of adolescent-friendly services, contraceptive provision in schools is still not allowed [20–22], suggesting the need for a policy framework that builds community support for adolescent contraceptive provision and use in all settings, including schools [23]. These findings highlight the need for further discussions and possible readjustment of policy on FP/C provision in schools as adolescents are unable to freely access these services at health facilities due to stigma and negative provider attitudes.

Stock-out of preferred methods affects demand and sustained use of contraceptives. Gaps in commodity supply have also been reported elsewhere [23–25], and are said to be as a result of the mismatch between supply and demand projections on most occasions [26]. Reducing incidences of contraceptive stock-outs will require strengthened commodity supply chain management, and balancing of demand projections and real-time availing of commodities [26, 27].

Experiences with contraceptive side effects shape choices and sustained use of methods [28–33]. Side effects have also been reported to perpetuate myths and misconceptions [34, 35]. For instance, inability to experience periods is associated with blood accumulation in the womb, leading to cancer or fibroids. Adopting innovative and context-specific community engagement strategies that aim at neutralising myths and rumours, and provide detailed information about side effects, may help address this barrier.

### Enablers to family and contraceptive services provision and utilisation

The study findings demonstrate that couples counselling services targeting male involvement in contraceptive choices are important enablers to contraceptive services provision and use. Such counselling services allow for increased male participation and support for family planning and contraceptive choices. Various studies have underlined that male partners are key decision makers [36, 37]. Hence couples counselling services help to educate and encourage male partners to support their spouses in using FP/C services [27].

Availability of personnel to offer a minimum method mix (barrier, short and medium acting) is an enabler, as it provides for choice among clients. However, the results also show limited LARC options in some health facilities due to non-training of their staff in this particular method. Strengthening of District level training and mentorship of personnel in LARC services at all health facilities will help improve access to this method.

Though found to be an enabler, integration of SRH interventions into community health systems remains complex [38], due to diverse norms, values, as well as the less formal mechanisms which shape coordination, accountability, health practice and health-seeking behaviour [39]. Key factors to consider at the community level may include the community’s capacity to engage and participate in the implementation process, commit and sustain health actions and ensure the development of effective partnerships between a complex array of actors involved in the intervention [22].

The findings also reveal high levels of knowledge about FP/C methods/services, which is consistent with the Zambia Demographic Health Survey (ZDHS) findings [10]. The desire to delay pregnancy is another community level enabler. Though high knowledge levels and desire to delay pregnancy provide an enabling environment for FP/C methods/services provision, they do not necessarily translate into utilisation, as evidenced by the low national CPR of 47% and high TFR of 5.3 births per woman [10].

The need to address some of the barriers suggested above cannot be overemphasised if we are to improve FP/C services practices among community members. Indeed, involving key community stakeholders (parents, teachers, churches and HCPs) in identifying, planning, implementation, monitoring and evaluation of FP/C services interventions, as proposed by the UPTAKE project, will contribute to improved service delivery and community support for contraceptives use. Furthermore, Interventions targeting provision of contraceptives in settings other than family planning clinics, strengthened integration of FP/C services, and training that addresses communication, counselling skills and cultural values clarification will be crucial to enhancing contraceptive use [23, 27, 40].

### Strengths and limitations

The collection of data from various categories of community members, HCPs and key stakeholders enabled gathering of a wide range of views, which allowed for strengthened data triangulation on key thematic areas. Community perspectives on barriers/enablers to FP/C methods/services uptake are not well explored, so this study adds to this perspective. Additionally, the qualitative team comprised professionals from various academic backgrounds, which further strengthened critical analysis and interpretation of the data.

However, there were also limitations to this research. Conducting the study in one setting/district per country, the use of a small sample of respondents, as well as, using only qualitative approaches, limits the generalisability of study findings. Though generalisability was not the intention, the rich description of the phenomena (community and health systems barriers and enablers to contraceptive provision and use), led to an in-depth account of barriers/facilitators to FP/C services in Kabwe district of Zambia. We believe this provides a valuable contribution to the body of knowledge on FP/C services provision and use in Low and Middle-Income settings.

## Conclusion

Our findings highlight community and health systems factors that ought to be considered by policy, planners and implementers in the design of FP/Cs programmes, to ensure sustained uptake and increased met needs. Tackling health systems inhibiting factors such as negative provider attitudes, gaps in sustained commodity and services supply, together with enforcing adolescent friendly policies will be vital in improving contraceptive uptake. Addressing community-level factors such as stigma, myths and negative beliefs will require full engagement of community members in service provision, which will also enhance community participation and support for FP/C services programmes. Additionally, FP/C services programmes should seek to build on the reported enabling factors both at community and health system levels for successful implementation.

Identifying the factors that shape delivery and adoption of FP/C interventions at various levels, and the role they play, both as facilitators and barriers, is important if we are to increase met needs for contraception. Understanding these factors forms the basis for future FP/C services programme planning and implementation efforts. We therefore recommend that future research not only identifies barriers and enablers to contraceptive provision and use, but also, goes further to explore approaches to maximise implementation success of community and health system participatory interventions seeking to increase met needs for contraception.

## Additional files

Additional file 1: Appendix D, FGD guide_females. (DOCX 44 kb)

Additional file 2: Appendix D, FGD guide_healthcare providers. (DOCX 45 kb)

Additional file 3: Appendix D, FGD guide_males. (DOCX 43 kb)

Additional file 4: Appendix D, In-depth interview guide_key stakeholders. (DOCX 51 kb)

## Abbreviations

CBDs: Community based distributors
CPR: Contraceptive prevalence rate
DHO: District health office
FGDs: Focus group discussions
FP/C: Family planning/contraceptives
HCPs: Healthcare providers
IDIs: In-depth interviews
NGO: Non-governmental organisation
NHC: Neighbourhood health committee
OPD: Outpatient department
PTMCT: Prevention of mother to child transmission
SDGs: Sustainable development goals
SMAGs: Safe motherhood action groups
SRH: Sexual and reproductive health
STIs: Sexually transmitted infections
TBAs: Traditional birth attendants
TFR: Total fertility rate
WHO: World Health Organisation
