# Supplementary material for: Community and health systems barriers and enablers to family planning and contraceptive services provision and use in Kabwe District, Zambia
Source: BMC Health Serv Res. 2018 May 31;18:390. doi: 10.1186/s12913-018-3136-4 (PMC5984360; doi:10.1186/s12913-018-3136-4)
Supplement: Supplementary file 2 — Appendix D, FGD guide_healthcare providers. (DOCX 45 kb) [file 12913_2018_3136_MOESM2_ESM.docx]

**Appendix D: Forms and Guides**

**UPTAKE Project**

**Guide 2.3: Focus group discussion guide (Health care providers)**

| **FOCUS GROUP ID NUMBER:** | **LOCATION OF FGD:** | **DATE (DD/MMM/YY):** | **START TIME:** | **END TIME:** | **MODERATOR INITIALS:** |
| --- | --- | --- | --- | --- | --- |

Introduction

We welcome all of you to this discussion. We are happy that you have spared some time to come and have this discussion. Let us start first by introducing ourselves. My name is …..and I will be leading the discussion. With me is…………………….. who will be writing down some notes

*[Read to participant]*

You have been invited here today to talk about the UPTAKE Project.

*Purpose*

We are interested in all your experiences, ideas, comments, suggestions and recommendations. This research is to help us understand how to best engage health care providers like yourself and know the experiences and challenges you as health care providers face in providing family planning and contraceptive services and information to your clients. This will help in sending feedback to policy makers and also be used to improve health services by identifying what you think are the challenges to provision of family planning and contraceptive services and information. All information will be treated with confidentiality.

*Explain the ground rules for discussion*

This is a friendly discussion so; there is no right or wrong answer. Everyone should relax and feel free to discuss his/her opinion. We would like to have one person talk at a time and when one person is talking, there will be no interruption until the person has ended then the next person will be allowed to air his/her view. There will be no side discussions. Anyone can contribute to the discussion at any time. Every one of you should feel free to disagree or agree in a cordial manner. Please remember that what we discuss here today is confidential, do not discuss private information disclosed in this group with others outside of this group. Please turn cell phones on to silent so as not to disrupt the discussion. We will spend about one and a half to two hours for the discussion and some refreshment will be served at the end of the discussion.

We have just reviewed the consent form, which describes the study in detail and gives us permission to speak with you. You are not required to answer all of my questions, and you may skip any questions. As a reminder, we will use a digital recorder to record our conversation.

Do you have any questions before we begin the discussion?

*[Turn on digital recorder.]*

I am (MODERATOR NAME) interviewing (FOCUS GROUP ID#) on [DATE] [START TIME]

|  | **Main question** | | | **Probe** |
| --- | --- | --- | --- | --- |
| **Family planning knowledge, attitudes and practices** | | | | |
| 1.1 | | Please describe your understanding of family planning (or contraception) services. | | - 1. Describe the different family planning/contraceptive methods you know about? *Probe for different methods.*   2. In your opinion, how well or poorly do family planning/contraceptive methods work to prevent pregnancy?   3. Who do you think should use family planning/contraceptive services? *Probe for marital status, parity, age (including teenagers), etc.*   4. What do women and girls like about using family planning/contraceptives? (Apart from preventing pregnancy, do they have other positive effects?) *Probe for specific advantages of different methods.*   5. What do women and girls not like about using family planning/contraceptives? Why? *Probe for specific things they don’t like about different methods?*   *Explore issues related to gender and perceived benefits/disadvantages.*   - 1. When choosing their family planning/contraceptive method, do people think about whether it prevents STIs/HIV? |
| 1.2 | | Do you provide contraceptive and family planning services in your workplace? | | *Yes/No.*  *Explore what services are available.*   1. Please describe your responsibilities and day-to-day work. 2. What role do you play in providing contraceptive/family planning services? 3. Please describe your family planning and contraceptive counselling procedures. 4. Do your facilities have a separate section for providing family planning/contraceptive services? Or is this service offered across all sections? 5. How often do women change their family planning/contraceptive methods used? *Explore why and how this is done?* |
| 1.3 | | What does your community know about family planning and contraceptive methods? | | - 1. What is/are the most common method(s) of family planning/contraception used in your community?   2. Why do you think this is/are the most common method(s)? |
| 1.4 | | What family planning/contraceptive methods are available in your community? | | - 1. What things make it difficult for young women to get and use family planning/contraceptive methods to prevent pregnancy when they want to use them?   *Probes: things about health services and health workers; other people’s opinions about young women using family planning/contraception (especially teenagers and unmarried women); whether or not people already have children; male partners’ opinions, etc.*   - 1. Are family planning/contraceptive services freely available at your facilities? |
| 1.5 | | How do women access family planning and contraceptive services in your community? | | *Explore where, how they get the method, travel requirements, who they get them from?*  *Give each participant a paper and a pen and ask them:*  Please draw a map of where family planning and contraceptive services are available in your communities. |
| 1.6 | | Who are the most important people in supporting women and girls in choosing and using family planning and contraceptive methods? | | *Probe for*   - Partner - Friends - Parents - Health workers - Community leaders - Religious leaders   *Explore why these people are the most important.* |
| 1.7 | | How comfortable are you counselling about family planning methods? | | 1. Why? 2. Who are you most comfortable to counsel? *Probe for age, sex, marital status, etc.* |
| **Health services capacity** | | | | |
| 2.1 | | What is the capacity of your facilities to provide family planning/contraceptive services? | 1. Do they have sufficient resources? 2. Please explain who is on staff at your facilities: how many doctors, nurses, others? 3. On an average day, how many clients are seen at the facilities? 4. How many clients come to your facilities for family planning and contraceptive services? 5. What community/communities are served by your health facilities? 6. What is the average time of a routine client visit for family planning/contraceptive services? | |
| 2.2 | | What role do you think healthcare providers play in assisting young people to access contraceptive/family planning methods? | 1. What role do you think they should play? 2. Do you think that the family planning/contraceptive needs of the young people are met by the healthcare providers? 3. Do you think that healthcare providers and young people have the same goals/vision for providing and accessing contraceptive/family planning services? | |
| 2.3 | | Who are the major clients in your community who access family planning/contraceptive services? | *Explore categories such as age, sex, marital status, rural vs urban, etc.* | |
| 2.4 | | What barriers and enablers are there to providing family planning and contraceptive services in your health facilities? | *Explore barriers and enablers.*  *Probe on available resources, number of staff, operation hours, number of rooms available vs number of clients attending the facility, waiting time, etc.* | |
| 2.5 | | Are there any religious or cultural barriers or facilitators to accessing family planning/contraceptive services? | *Explore barriers and facilitators at both religious and cultural level.*   1. *If barriers were reported:*   How can these be overcome? | |
| 2.6 | | How do you think decentralisation of services has affected (or could affect) community access to contraceptive/ family planning services? | *Explore both positive and negative outcomes.* | |
| 2.7 | | Are there any special services for women and girls wanting family planning and contraceptive services at your health facilities or in the local community? | *Explore what these services are, and where they are.*  *If there are services, explore their accessibility and whether they are used or not.* | |
| 2.8 | | What, if any, unique needs do women and girls in your community have in the context of accessing family planning and contraceptive services? |  | |
| **Quality of care** | | | | |
| 3.1 | | How would you define good quality family planning/contraceptive services? | | What constitutes good quality of care? |
| 3.2 | | Some people say quality of care is influenced by issues such as available health care workers, integration of services, facility operation hours, number of rooms available, number of clients attending the facility, waiting time, etc. Which of these are important for you in describing good quality care? | |  |
| 3.3 | | Are quality family planning/contraceptive services available to people in your community? | | *Yes/No.*   1. Are your health care facilities providing good quality family planning/contraceptive services? *Yes/No.*   *Explore using next question.* |
| 3.4 | | How could your facilities provide better quality family planning/contraceptive services? | | 1. How do you think the services should be delivered? 2. Who should deliver the services? 3. What other information should be given to clients about family planning/contraceptive services? |
| **Community participation and relationships** | | | | |
| 4.1 | Community members and groups participate in different ways within the health system. How would you define community participation in this community? | | | *Explore group understanding vs individual opinions?* |
| 4.2 | What are some of the existing community participation activities in this area? | | | 1. Who participates in these activities? And how? 2. How does the community feel about these activities? 3. What community participation activities work and which ones don’t work? *Explore why– probe for issues of age, religion and cultural acceptability of community participation.* 4. What are some of the challenges to community participation in your area? *Also explore if no community participation activities in the area.* 5. Who should participate if a project is created on family planning and contraceptive service in this community? How should they participate? |
| 4.3 | How do you engage with the community about family planning/contraceptive services? | | | 1. How do you, as health care providers, feel about engaging with the community about family planning/contraceptive services? |
| 4.4 | How do health care providers take community voices about family planning/contraceptive services into account? | | | *Explore.*   1. Who initiates discussions on family planning/contraception? (Clients vs health care providers) |
| 4.5 | Do you have links with other health care providers in the community? | | | 1. Describe these relations/links. |
| 4.6 | How do you think community participation can be used to improve access to family planning/contraceptive services? | | | What are your recommendations for improving community engagement with health care providers when accessing family planning/contraceptive services?  *Probe for consideration of age (teenagers vs older women), married vs unmarried, rural vs urban, women with or without children, etc.* |
| 4.7 | What role do you think the community should play to improve future access to family planning/contraceptive services? | | | 1. How can the community be engaged in future interventions for improved uptake of family planning/contraceptive services? 2. What could these interventions be? 3. *Explore.* |
| **Conclusion** | | | | |
| 5.1 | Do you have anything else that you would like to tell us about family planning/contraception and community participation before we end? | | |  |

This is the end of our discussion. Thank you for your time.
