# Supplementary material for: Community and health systems barriers and enablers to family planning and contraceptive services provision and use in Kabwe District, Zambia
Source: BMC Health Serv Res. 2018 May 31;18:390. doi: 10.1186/s12913-018-3136-4 (PMC5984360; doi:10.1186/s12913-018-3136-4)
Supplement: Supplementary file 3 — Appendix D, FGD guide_males. (DOCX 43 kb) [file 12913_2018_3136_MOESM3_ESM.docx]

**Appendix D: Forms and Guides**

**UPTAKE Project**

**Guide 2.2: Focus group discussion guide (Males)**

| **FOCUS GROUP ID NUMBER:** | **LOCATION OF FGD:** | **DATE (DD/MMM/YY):** | **START TIME:** | **END TIME:** | **MODERATOR INITIALS:** |
| --- | --- | --- | --- | --- | --- |

*[Read to participant]*

You have been invited here today to talk about the UPTAKE Project.

*Purpose*

We are interested in all your experiences, ideas, comments, suggestions and recommendations. This research is to help us understand how to best engage community members like yourself and know the experiences and challenges the community faced and are still facing in accessing family planning and contraceptive services. This will help in sending feedback to policy makers and also be used to improve health services by identifying what you think are the challenges to accessing family planning and contraceptive services. All information will be treated with confidentiality.

*Explain the ground rules for discussion*

This is a friendly discussion so; there is no right or wrong answer. Everyone should relax and feel free to discuss his opinion. We would like to have one person talk at a time and when one person is talking, there will be no interruption until the person has ended then the next person will be allowed to air his view. There will be no side discussions. Anyone can contribute to the discussion at any time. Every one of you should feel free to disagree or agree in a cordial manner. Please remember that what we discuss here today is confidential, do not discuss private information disclosed in this group with others outside of this group. Please turn cell phones on to silent so as not to disrupt the discussion. We will spend about one and a half to two hours for the discussion and some refreshment will be served at the end of the discussion.

We have just reviewed the consent form, which describes the study in detail and gives us permission to speak with you. As a reminder, you are not required to answer all of my questions, and you may skip any questions. As a reminder, we will use a digital recorder to record our conversation.

Do you have any questions before we begin the discussion?

*[Turn on digital recorder.]*

I am (MODERATOR NAME) interviewing (FOCUS GROUP ID#) on [DATE] [START TIME]

|  | **Main question** | **Probe** |
| --- | --- | --- |
| **Family planning knowledge, attitudes and practices** | | |
| 1.1 | Please describe your understanding of family planning (or contraception) services. | - 1. Describe the different family planning/contraceptive methods you know about. *Probe for different methods.*   2. In your opinion, how well or poorly do family planning/contraceptive methods work to prevent pregnancy?   3. Who do you think should use family planning/contraceptive services? *Probe for marital status, parity, age (including teenagers), etc.*   4. What do men like about family planning/contraceptives? (Apart from preventing pregnancy, do they have other positive effects?) *Probe for specific advantages of different methods.*   5. What do men not like about family planning/contraceptives? Why? *Probe for specific things they don’t like about different methods.*   *Explore issues related to gender and perceived benefits/disadvantages.*   - 1. When choosing their family planning/contraceptive method, do people think about whether it prevents STIs/HIV? |
| 1.2 | What is/are the most common method(s) of family planning/contraception used in your community? | - 1. Why do you think this is the most common method(s)?   2. Which is your and your partner’s preferred method and why?   *If participants and their partners don’t use family planning/ contraception, explore why.*  *Facilitator to make a note of numbers who don’t use family planning/contraception methods.* |
| 1.3 | What family planning/contraceptive methods are available in your community? | - 1. What things make it difficult for young people to get and use family planning/contraceptive methods to prevent pregnancy when they want to use them?   *Probes: things about health services and health workers; other people’s opinions about young women using family planning/contraception (especially teenagers and unmarried women); whether or not people already have children; male partners’ opinions, etc.*   - 1. Are family planning/contraceptive services freely available at your clinic? |
| 1.4 | Is there a family planning/contraceptive method you and your partner wish to try but it is not available at your clinic? | *Yes/No?*  *Moderator to ask each participant, and record responses.*  *Explore what these methods are.* |
| 1.5 | Who are the most important people in supporting women and girls in choosing and using family planning and contraceptive methods? | *Probe for*   - Partner - Friends - Parents - Health workers - Community leaders - Religious leaders   *Explore why these people are the most important.*   1. Do you discuss family planning/contraception with your partner(s)? 2. Who makes the decision about family planning/contraception in your relationships? |
| **Barriers and enablers to family planning access** | | |
| 2.1 | Women, girls and families go through different experiences using family planning and contraceptive methods. What are some of the experiences you or your friends have encountered? | 1. Do women and their partners change family planning/contraceptive methods used? *Explore why, and what methods.* |
| 2.2 | Can we discuss your experiences accessing family planning and contraceptive services?  *[Note to interviewer: participants may or may not have experiences accessing these services, and some may be limited to condom access, but allow participants to express their views without leading them].* | 1. How did you know about the method/s available? 2. Did you seek out these family planning/contraceptive services, or were you approached to use these services? 3. How was family planning/contraception approached and discussed with you? *[if participant refers to healthcare provider, explore questions in 2.3 below]* 4. Who are your main sources of health information about family planning/contraceptive use, risks?   *Give each participant a paper and pen, ask participants:*  Please draw a picture of where you/your partner access your family planning/contraceptive services from, in relation to your home. Include estimates of distance/time taken to get to the facilities. |
| 2.3 | Can we discuss your experiences with health care providers when seeking or being spoken to about family planning/contraceptive services?  *[Note to interviewer: Participants may not have experiences with healthcare providers, if not, do not focus on probes].* | *Explore attitude of healthcare providers during visit.*   1. Did you initiate the discussion of family planning/contraceptive services? How did the health care provider react? 2. If the healthcare provider initiated the discussion, how did they go about it? 3. Did you ask questions and how did the health worker respond to your queries? 4. Were your concerns effectively addressed by the health care provider? |
| 2.4 | Are there any religious or cultural barriers or facilitators to accessing family planning/contraceptive services? | *Explore barriers and facilitators at both religious and cultural level.*   1. *If barriers were reported:*   How can these be overcome? |
| 2.5 | What role do you think healthcare providers play in assisting young people to access contraceptive/family planning methods? | 1. What role do you think they should play? 2. Do you think that the family planning/contraceptive needs of the young people are met by the healthcare providers? 3. Do you think that healthcare providers and young people have the same goals/vision for providing and accessing contraceptive/family planning services? |
| 2.6 | What advice would you give someone else who is not using a contraceptive/family planning method or who may be interested in using or changing methods? | *Probe for each:*   1. *Someone who is not using a method* 2. *Someone who is interested in changing methods* |
| 2.7 | What resources are available in your community to support women and girls in accessing family planning and contraceptive services? |  |
| **Quality of care** | | |
| 3.1 | How would you define good quality family planning/contraceptive services? | What constitutes good quality of care? |
| 3.2 | How would you like to receive quality family planning/contraceptive services? | 1. How would you like to access the services? 2. Where would you like to access the services from? 3. Who would you like to access the services from? 4. What other information do you need about family planning services? |
| 3.3 | Are quality family planning/contraceptive services available to people in your community? | Explore why or why not? |
| **Community participation** | | |
| 4.1 | Community members and groups participate in different ways within the health system. How would you define community participation in this community? | *Explore group understanding vs individual opinions?* |
| 4.2 | What are some of the existing community participation activities in this area? | 1. Who participates in these activities? And how? 2. How does the community feel about these activities? 3. What community participation activities work and which ones don’t work? *Explore why– probe for issues of age, religion and cultural acceptability of community participation.* 4. What are some of the challenges to community participation in your area? *Also explore if no community participation activities in the area.* 5. Who should participate if a project is created on family planning and contraceptive service in this community? How should they participate? |
| 4.3 | How do you think community participation can be used to improve access to family planning/contraceptive services? | What are your recommendations for improving community engagement with healthcare providers when accessing family planning/contraceptive services?  *Probe for consideration of age (teenagers vs older women, married vs unmarried, rural vs urban, etc.)* |
| 4.4 | What role do you think the community should play to improve future access to family planning/contraceptive services? | 1. How can the community be engaged in future interventions for improved uptake of family planning/contraceptive services? 2. What could these interventions be? 3. *Explore.* |
| **Conclusion** | | |
| 5.1 | Do you have anything else that you would like to tell us about family planning/contraception and community participation before we end? |  |

This is the end of our discussion. Thank you for your time.
