# Supplementary material for: Community and health systems barriers and enablers to family planning and contraceptive services provision and use in Kabwe District, Zambia
Source: BMC Health Serv Res. 2018 May 31;18:390. doi: 10.1186/s12913-018-3136-4 (PMC5984360; doi:10.1186/s12913-018-3136-4)
Supplement: Supplementary file 4 — Appendix D, In-depth interview guide_key stakeholders. (DOCX 51 kb) [file 12913_2018_3136_MOESM4_ESM.docx]

**Appendix D: Forms and Guides**

**UPTAKE Project**

**Guide 1: In-depth interview guide (Key stakeholders)**

| **PID NUMBER:** | **LOCATION OF IDI:** | **DATE (DD/MMM/YY):** | **START TIME:** | **END TIME:** | **INTERVIEWER INITIALS:** |
| --- | --- | --- | --- | --- | --- |

*[Read to participant]*

You have been invited here today to talk about the UPTAKE project.

Purpose:

I am interested in all your experiences, ideas, comments, suggestions and recommendations. This research is to help me understand how to best engage community members and know the experiences and challenges women and girls in the community face in accessing family planning and contraceptive services. This will help in sending feedback to policy makers and also be used to improve health services by identifying what you think are the challenges to accessing family planning and contraceptive services. All information will be treated with confidentiality.

Explain the ground rules for the interview:

We have just reviewed the consent form, which describes the study in detail and gives us permission to speak with you., You are not required to answer all of my questions, and you may skip any questions. As a reminder, I will use a digital recorder to record our conversation.

Do you have any questions before we begin the interview?

*[Turn on digital recorder.]*

I am (INTERVIEWER NAME) interviewing (PARTICIPANT ID#) on [DATE] [START TIME]

| 1. **Background**   *[Please complete information in spaces below]* | | |
| --- | --- | --- |
| 1. | Sex: | Male Female |
| 2. | Sector representing: |  |
| 3. | Job title: |  |
| 4. | Office/Department: |  |
| 5. | Highest educational level: |  |
| 6 | Number of years in current position: |  |
| 7. | What are your primary responsibilities? |  |
| 8. | Age at last birthday | ___________ (age in years) |

|  | **Main questions** | **Probes** | |
| --- | --- | --- | --- |
| **Family planning knowledge, attitudes and practices** | | | |
| 9.1 | Please describe your understanding of family planning/ contraceptionservices. | - 1. Describe the different family planning/contraceptive methods you know about.   2. *Probe for different methods.* | |
| 9.2 | What is/are the most common method(s) of family planning/contraception used in your community? | - 1. Why do you think this is the most common method(s)?   2. Do women and their partners change family planning/contraceptive methods?   *Explore why, what methods and frequency of change.* | |
| 9.3 | What family planning/contraceptive methods are available (offered) in your community? | - 1. What things make it difficult for people to get and use family planning/contraceptive methods?   *Probes: things about health services and health workers; other people’s opinions about why certain people should/should not usefamily planning/contraception (especially teenagers and unmarried); whether or not people already have children; male partners’ opinions; culture, religion; etc.* | |
| 9.4 | Who are the key people that support women and girls in choosing and using family planning and contraceptive methods? | *Probe for*   - Partner - Friends - Parents - Health workers - Community leaders - Religious leaders   *Explore why these people are the most important.*   1. Who makes the decision about using family planning/contraception in your community?   *Explore issues related to gender and power relations in family planning/contraceptive decision making.* | |
| **Barriers and enablers to family planning/contraceptive access** | | | |
| 10.1 | Women, girls and families go through different experiences accessing family planning and contraceptive methods.  What are the experiences of your community members in accessing these services? | | 1. What are some of the barriers and enablers to accessing family planning/contraceptive services in your area?   *Probe on:*   - access to healthcare facilities with family planning/contraceptive services, - capacity of healthcare facilities, - attitudes and knowledge of healthcare providers, - availability of information about contraceptive/family planning services, - culture, traditions, religion; etc. |
| 10.2 | What are the major sources of health information about family planning/contraceptive services in the community? | | 1. Where does the community access their information about family planning and contraceptive services? |
| 10.3 | Do the healthcare facilities in your area have the capacity to provide family planning/contraceptive services? | | *Probe on available resources, number of staff, operation hours, number of rooms available vs number of clients attending the facility, waiting time, etc.*   1. Do healthcare providers in your area have capacity to provide family planning/contraceptive services to all potential clients?   *Explore training received, number of years of practical experience, etc.* |
| 10.4 | How would you describe the knowledge and attitudes of healthcare providers towards providing family planning and contraceptive services to their clients? | | *Explore attitudes towards different categories of clients, including: Age of clients, marital status, sex of clients, occupation, rural vs urban, etc.* |
| 10.5 | Where do you think family planning/contraceptive services should be made available to members of the community? | | *Probe for facility type, service area, etc.*   1. Who do you think should be providing family planning/contraceptive services to the community? |
| 10.6 | Who are the major clients in your community who access family planning/contraceptive services? | | *Explore categories such as age, sex, marital status, rural vs urban, etc.* |
| 10.7 | What role do you think healthcare providers play in assisting young people to access contraceptive/family planning methods? | | 1. What role do you think they should play? 2. Do you think that the family planning/contraceptive needs of the young people are met by the healthcare providers? 3. Do you think that healthcare providers and young people have the same goals/vision for providing and accessing contraceptive/family planning services? |
| 10.8 | What advice would you give someone who is not using a contraceptive/family planning method or who may be interested in using or changing methods? | | *Probe for each:*   1. Someone who is not using a method 2. Someone who is interested in changing methods |
| 10.9 | What resources are available in your community to support women and girls in accessing family planning and contraceptive services? | | 1. Are there any special services for women and girls wanting family planning and contraceptive services at your health facilities or in the local community?   *Explore what these services are, and where they are.*  *If there are services, explore their accessibility and whether they are used or not.* |
| 10.10 | How do you think decentralisation of services has affected (or could affect) community access to contraceptive/ family planning services? | | *Explore both positive and negative outcomes.* |
| **Quality of care** | | | |
| 11.1 | How would you define good quality family planning/contraceptive services? | | 1. What constitutes good quality of care? |
| 11.2 | Some people say quality of care is influenced by issues such as available healthcare workers, integration of services, facility operation hours, number of rooms available, number of clients attending the facility, waiting time, etc. Which of these are important for you in describing good quality care? | |  |
| 11.3 | Are quality family planning/contraceptive services available to people in your community? | | *Explore why or why not?* |
| 11.4 | How do you think healthcare facilities could provide quality family planning/contraceptive services? | | 1. How do you think the services should be delivered? 2. Who should deliver the services? 3. What other information should be given to clients about family planning/contraceptive services? |
| **Community participation** | | | |
| 12.1 | Community members and groups participate in different ways within the health system. How would you define community participation in this community? |  | |
| 12.2 | What are some of the existing community participation activities in this area? | 1. Who participates in these activities? And how? 2. How does the community feel about these activities? 3. What community participation activities work and which ones don’t work? *Explore why – probe for issues of age, religion and cultural acceptability of community participation.* 4. What are some of the challenges to community participation in your area? (*Also explore if no community participation activities in the area).* 5. Who should participate if a project is created on family planning and contraceptive service in this community? How should they participate? | |
| 12.3 | What role do you play in facilitating community participation in your area? | 1. How does your role in the community relate to health and family planning/contraceptive services access for the community? | |
| 12.4 | How do you think community participation can be used to improve access to family planning/contraceptive services? | 1. What are your recommendations for improving community engagement with healthcare providers when accessing family planning/contraceptive services? 2. *Probe for consideration of age (teenagers vs older women, married vs unmarried, rural vs urban, etc.)* | |
| 12.5 | What role do you think the community should play to improve future access to family planning/contraceptive services? | 1. How can the community be engaged in future interventions for improved uptake of family planning/contraceptive services? 2. What could these interventions be? 3. *Explore.* | |
| **Conclusion** | | | |
| 13.1 | Do you have anything else that you would like to tell us about family planning/contraception and community participation before we end? |  | |

This is the end of our discussion. Thank you for your time.
